# Electrical Properties of CZO Films Prepared by Ultrasonic Spray Pyrolysis

**DOI:** 10.3390/ma7117304

**Published:** 2014-11-05

**Authors:** Lung-Chien Chen, Cheng-An Hsieh, Xiuyu Zhang

**Affiliations:** Department of Electro-Optical Engineering, National Taipei University of Technology, No. 1, Section 3, Chung-Hsiao E. Road, Taipei 106, Taiwan; E-Mails: t102658033@ntut.edu.tw (C.-A.H.); t101658072@ntut.edu.tw (X.Z.)

**Keywords:** CZO, XRD, photoluminescence, Raman analysis

## Abstract

CuZnO (CZO) films have attracted increasing amounts of attention due to their promising potential applications in semiconductor devices. ZnO shows n-type conductivity, and attempts have been made to dope several elements in ZnO to improve the electrical properties. This study investigated the electrical property transitions of CZO films and determined the copper concentration at which the conductivity of CZO films will change from n-type to p-type. In this study, CZO films were fabricated by ultrasonic spray pyrolysis with copper acetate, zinc acetate, and ammonium acetate precursor solution. The concentrations of Cu ions in the CZO films were controlled by the concentration ratios of copper acetate to zinc acetate in the precursor solutions. In addition, these samples were analyzed by Hall effect measurements, X-ray diffraction, transmittance measurements, and photoluminescence measurements. The results show that the conductivity of the CZO film changes from n-type to p-type when the copper ion concentration in the film is 5%.

## 1. Introduction

Zinc oxide (ZnO) is a popular material because of its large band gap (3.4 eV) and large exciton binding energy (60 meV) [1,2]. Due to their superior properties such as high crystalline quality, large aspect ratio, and quantum confinement effects [3,4], ZnO nanostructures have attracted great research interest. Different techniques such as molecular beam epitaxy, sputtering, sol-gel processing, vapor deposition, and electrochemical deposition have been employed to fabricate ZnO nanowires and nanorods, which have been widely used in laser devices, gas sensors, ultraviolet–visible light emission devices, and many other applications [5,6,7,8]. Ai *et al*. [9] have studied the room temperature ferromagnetism of n-type Cu-doped epitaxial ZnO films, while Aravind *et al*. [10] focused on the optical and magnetic properties of copper-doped ZnO nanorods; in addition, many studies have been conducted on CuZnO (CZO) [11,12,13,14,15].

ZnO shows n-type conductivity; attempts have been made to dope various elements including Cr, Co, Ni, Mn, or Fe in ZnO to improve its electrical properties [16,17,18,19,20,21]. This study focuses on Cu-doped ZnO because of its promising potential applications in semiconductor devices. Recently, doping with Cu atoms has attracted increasing attention due to the lack of clustering and the fact that the secondary phases of Cu and copper oxides (Cu_2_O and CuO) are not ferromagnetic (FM) [22].

Many different methods have been used to obtain Cu-doped ZnO; for example, Kim *et al*. [23] used RF magnetron sputtering, and Zheng *et al*. [24] used a sol-gel method. In this study, CZO thin films were fabricated on glass substrates using ultrasonic spray pyrolysis. It is important to know the properties of CuZnO thin films; this study investigated CZO thin films doped with different concentrations of Cu ranging from 0% to 6%. These samples were analyzed by Hall effect measurements, X-ray diffraction, transmittance measurements, and photoluminescence measurements.

## 2. Results and Discussion

### 2.1. Morphological and Structural Properties

**Figure 1:**
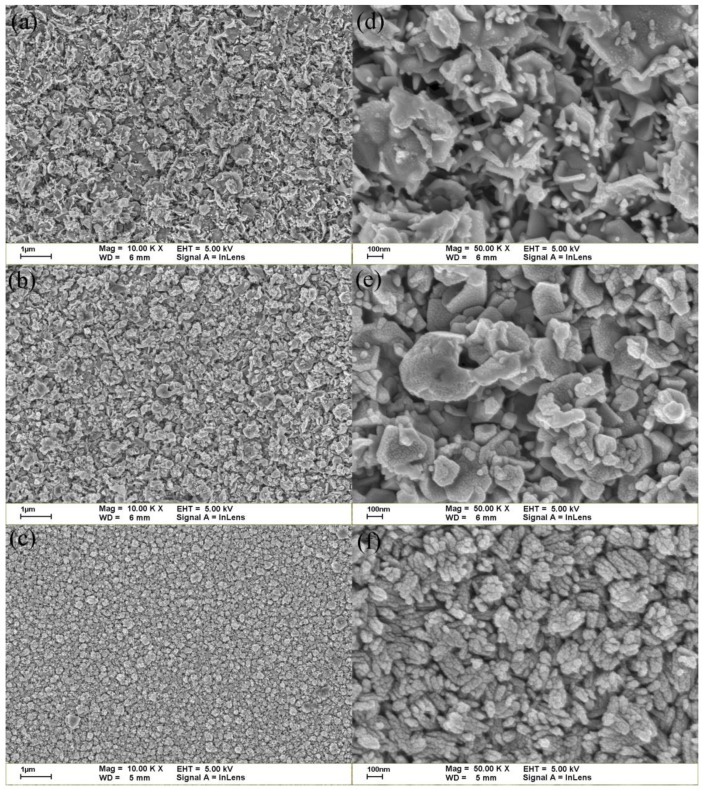
SEM images of (**a**) Zn_0.98_Cu_0.02_O; (**b**) Zn_0.96_Cu_0.04_O and (**c**) Zn_0.94_Cu_0.06_O. (**d**–**f**) show the images in (**a**–**c**) at five times greater magnification, respectively.

Figure 1 shows the surface morphologies of the CZO thin films with different Cu concentrations. As the Cu concentration increased, the films developed tightly packed grains and relatively smooth surfaces.

**Figure 2:**
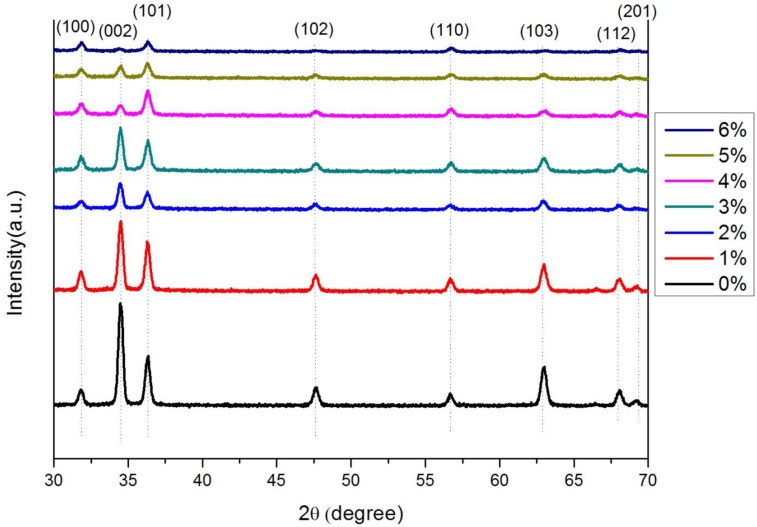
XRD patterns of Cu*_x_*Zn_1−*x*_O (*x* = 0–0.06).

Figure 2 shows the XRD patterns of samples with different Cu concentrations. A peak at a Bragg’s angle of ~34°, which is attributed to the ZnO (002) plane, is dominantly observed for all samples. The intensity of the ZnO (002) peak obviously decreased with increasing Cu concentration, which might be caused by lattice distortion due to the incorporation of Cu ions because the ionic radii of Zn^2+^ (0.60 Å) and Cu^2+^ (0.57 Å) ions are different [25]. Doping with Cu appears to decrease the crystallinity compared to pure ZnO films. Table 1 shows the Hall effect measurements of the CZO thin films. The natural ZnO thin film is n-type; however, the CZO thin film changed from n-type to p-type when the concentration of Cu was increased to 5%.

**Table 1:** Hall effect measurements of CuZnO (CZO) thin films.

Sample	Mobility (cm^2^/V·s)	Concentration (cm^−3^)	R_0_ (Ω·cm)	Conductivity
ZnO	6.96	1.347 × 10^17^	6.66	n
Cu_0.03_Zn_0.97_O	1.06	2.599 × 10^17^	22.76	n
Cu_0.04_Zn_0.96_O	0.79	3.016 × 10^18^	2.619	n
Cu_0.05_Zn_0.95_O	0.692	5.517 × 10^18^	1.634	p

### 2.2. Transmission and Absorption

**Figure 3:**
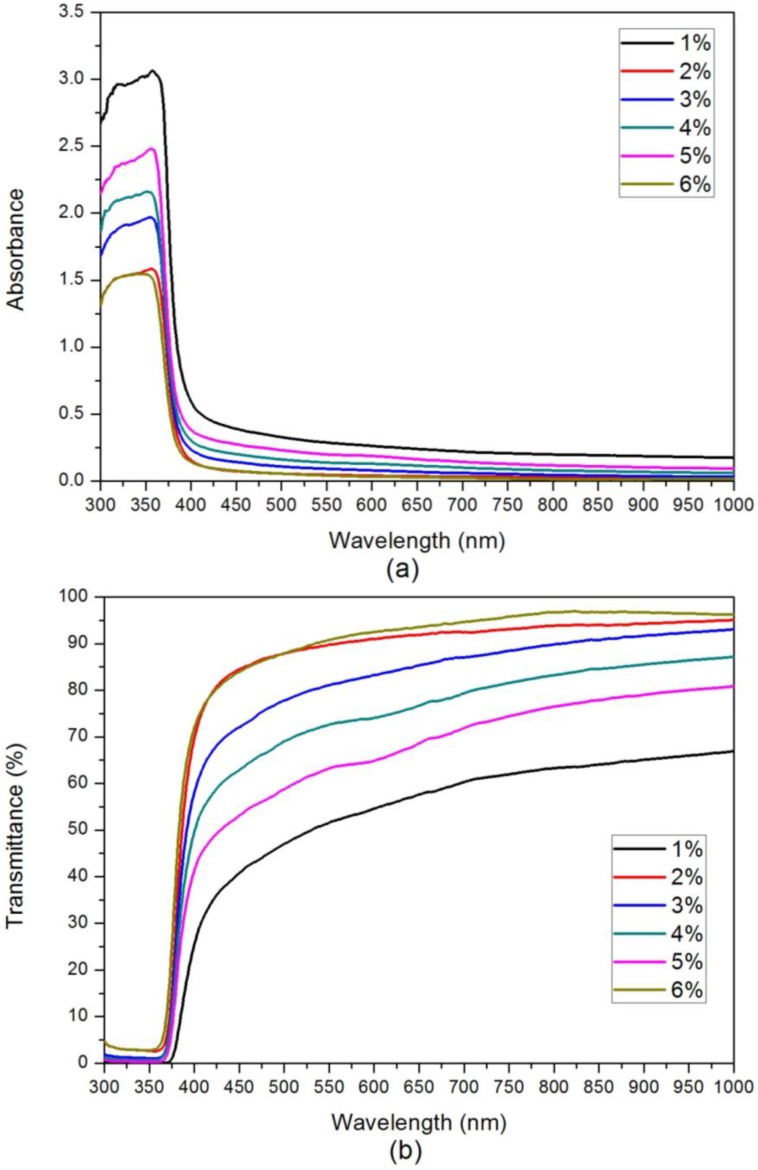
(**a**) Absorption curves and (**b**) optical transmittance spectra.

The results of UV-Visible absorption and transmittance measurements are shown in Figure 3. Figure 3a shows that the sharp UV absorption edges of all the CZO films are in the range of 385–410 nm. The absorbance was calculated by the following equation [26]:

*A* = 1 − *R*(*film*) − *T*(*film*)/*T*(*substrate*) (1)

where *R* and *T* are the measured reflectance and transmittance, respectively. Two main effects caused the exhibited absorption of the samples in the visible region: (1) strong d-p coupling between Cu and O upshifts the O_2p_ orbital and narrows the fundamental bandgap; and (2) the Cu_3d_ orbital creates impurity bands above the ZnO valance band. The band gaps of the CZO films with Cu concentrations ranging from 1% to 6% are 3.02 eV (Cu_0.01_Zn_0.99_O), 3.06 eV (Cu_0.02_Zn_0.98_O), 3.09 eV (Cu_0.03_Zn_0.97_O), 3.11 eV (Cu_0.04_Zn_0.96_O), 3.18 eV (Cu_0.05_Zn_0.95_O), and 3.22 eV (Cu_0.06_Zn_0.94_O). This demonstrates that increasing Cu concentration increased the energies of the absorption edge. Figure 3b shows that the film transmittances in the visible region are above 40%.

### 2.3. Raman Analysis

**Figure 4:**
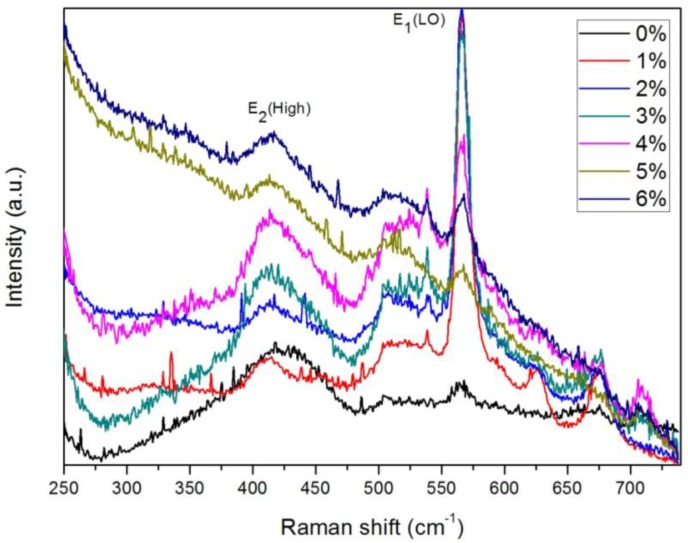
Raman spectra of Cu*_x_*Zn_1−*x*_O (*x* = 0–0.06).

Figure 4 shows the room temperature Raman spectra of the CZO thin films, demonstrating the influence of Cu-doping on the ZnO structure. The E_2_ (high) mode is characteristic of the wurtzite phase [27]; the intensity of the E_2_ peak increased with increasing Cu concentration. The spectra also indicate peak shifting from 425 cm^−1^ to 415 cm^−1^ with increasing Cu doping amount compared to the ZnO thin film; this frequency shift was caused by the lattice defects and lattice disorder [28,29]. The peak at about 566 cm^−1^ is assigned to the E_1_ longitudinal optical (LO) mode [E_1_(LO)] and was caused by oxygen vacancy and Zn interstitial defects [30,31,32].

### 2.4. Photoluminescence

**Figure 5:**
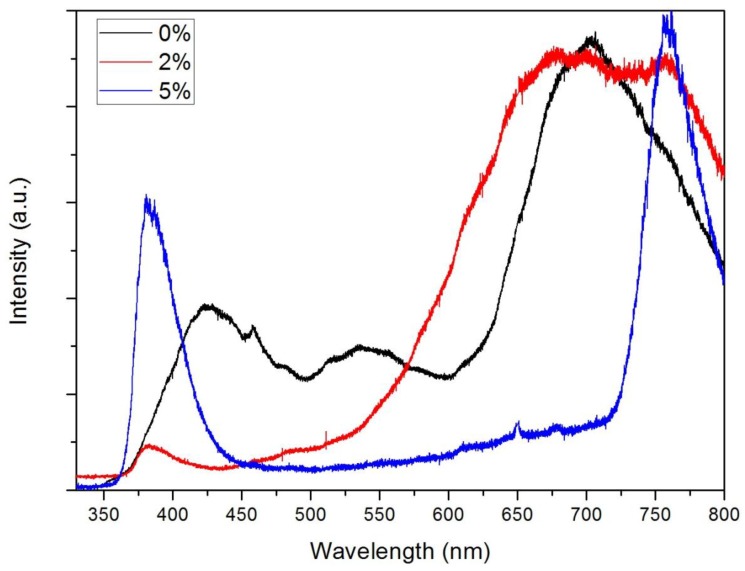
Photoluminescence spectra of Cu*_x_*Zn_1−*x*_O (*x* = 0, 0.02, 0.05).

A photoluminescence study was conducted to investigate the effect of Cu doping on the quality and wavelength of emission. Figure 5 shows the photoluminescence spectra of the CZO thin films. The peak at 424 nm is attributed to the wurtzite structure of ZnO. With Cu-doping, the peak shifted and an ultraviolet emission attributed to the near band-edge free-exciton transition appeared at 380 nm [33,34]. This result is in good agreement of the shift in absorption edge with increasing Cu concentration. A broad yellow-red emission from 600 nm to 750 nm is also observed [35] due to copper impurities or oxygen vacancies. Many intrinsic or extrinsic defects such as antisite oxygen, surface defects, and zinc vacancies have been reported in the visible region [36,37].

## 3. Experimental Section 

In this study, CuZnO (CZO) thin films were fabricated on glass substrates using ultrasonic spray pyrolysis with copper acetate [Cu(CH_3_COO)_2_·H_2_O], zinc acetate [Zn(CH_3_COO)_2_·2H_2_O], and ammonium acetate [CH_3_COONH_4_] precursor solution. The sizes of the glass substrates were 2.5 × 2.5 cm^2^. The glass substrates were cleaned using ultrasonic cleaner with acetone, ethanol, and pure water. Each cleaning process lasted for 5 min. A mixture of copper acetate, zinc acetate, and ammonium acetate powders were used to prepare the solution. The molar ratio of copper acetate mixed with zinc acetate to ammonium acetate was maintained at 1:3. In the mixed powder of copper acetate and zinc acetate, the molar concentration of copper acetate ranged from 0 to 0.06. The powders were put in 20 mL of pure water and mixing by magnetic stirrer for 1 h. After mixing, the solution was changed from liquid-state to mist-state by oscillation for 30 min. The CZO thin films were then fabricated using ultrasonic spray pyrolysis at 500 °C for 15 min; nitrogen gas was used in the pyrolysis process. After cooling to room temperature, the samples were analyzed by Hall-effect measurements, X-ray diffraction, and transmittance and photoluminescence measurements.

## 4. Conclusions

In this study, CZO thin films doped with different concentrations of Cu were investigated. The conductivity of the natural CZO thin film is n-type and changes to p-type when the doping concentration of Cu is 5%. As the Cu content of the CZO thin films increases from 1% to 6%, the film band gap increases from 3.02 eV to 3.22 eV. The Raman spectra show that the intensity of the E_2_ peak increases with increasing Cu concentration; compared to the ZnO thin film, the peak shifts from 425 cm^−1^ to 415 cm^−1^ due to lattice defects and lattice disorder. In addition, the E_1_ longitudinal optical (LO) mode is generated by oxygen vacancy and Zn interstitial defects. In photoluminescence peak shifts with increasing Cu concentration, resulting in an ultraviolet emission at 380 nm; this is attributed to the near band-edge free-exciton transition.

## References

[1] Hsiao C.S., Chen S.Y., Kuo W.L., Lin C.C., Cheng S.Y. (2008). Synthesis and optical properties of white-light-emitting alumina/ZnO nanotubes. Nanotechnology.

[2] Look D.C., Claflin B., Alivov Y.I., Park S.J. (2004). The future of ZnO light emitters. Phys. Status Solidi (a).

[3] Pauporté T., Lincot D., Viana B., Pellé F. (2006). Toward laser emission of epitaxial nanorod arrays of ZnO grown by electrodeposition. Appl. Phys. Lett..

[4] Law M., Greene L.E., Johnson J.C., Saykally R., Yang P. (2005). Nanowire dye-sensitized solar cells. Nat. Mater..

[5] Aoumeur F.Z., Benkabou K.H., Belgoumène B. (2003). Structural and dynamical properties of ZnO in zinc-blende and rocksalt phases. Phys. B Condens. Matter.

[6] Soudi A., Khan E.H., Dickinson J.T., Gu Y. (2009). Observation of unintentionally incorporated nitrogen-related complexes in ZnO and GaN nanowires. Nano Lett..

[7] Morkoç H., Strite S., Gao G.B., Lin M.E., Sverdlov B., Burns M. (1994). Large-band-gap SiC, III-V nitride, and II-VI ZnSe-based semiconductor device technologies. J. Appl. Phys..

[8] Bagnall D.M., Chen Y.F., Shen M.Y., Zhu Z., Goto T., Yao T. (1998). Room temperature excitonic stimulated emission from zinc oxide epilayers grown by plasma-assisted MBE. J. Cryst. Growth.

[9] Ai Z., Wu H., Lin Y., Zhou Z., Wang S., Liu C. (2012). Carrier concentration effect of Cu-doped ZnO films for room temperature ferromagnetism. Jpn. J. Appl. Phys..

[10] Aravind A., Jayaraj M.K., Kumar M., Chandra R. (2013). Optical and magnetic properties of copper doped ZnO nanorods prepared by hydrothermal method. J. Mater. Sci. Mater. Electron..

[11] Zhang L.Q., Ye Z.Z., Lu J.G., Lu B., Zhang Y.Z., Zhu L.P., Zhang J., Yang D., Wu K.W., Huang J. (2010). Influence of p-type and n-type dopants on the magnetic properties of ZnCuO based diluted magnetic semiconductor thin films. J. Phys. D Appl. Phys..

[12] Chan Y.-M., Wu Y.-T., Jou S. (2012). Oxide solar cells fabricated using zinc oxide and plasma-oxidized cuprous oxide. Jpn. J. Appl. Phys..

[13] Li M.-H., Chen X.-M., Xu J.-P., Zhang X.-S., Wu Y.-Y., Li P., Niu X.-P., Luo C.-Y., Li L. (2012). Synthesis and photoluminescent properties of ZnO:Cu/ZnO core/shell nanocrystals. Optoelectron. Lett..

[14] Xiong Z., Chen L., Zheng C. (2011). Theoretical studies on p-type conduction in (S,Cu) co-doped ZnO. Adv. Mater. Res..

[15] Liu H., Yang J., Hua Z., Zhang Y., Yang L., Xiao L., Xie Z. (2010). The structure and magnetic properties of Cu-doped ZnO prepared by sol-gel method. Appl. Surf. Sci..

[16] Sato K., Katayama-Yoshida H. (2002). Ab initio study on the magnetism in ZnO-, ZnS-, ZnSe- and ZnTe-based diluted magnetic semiconductors. Phys. Status Solidi B.

[17] Lee S., Shon Y., Lee S.-W., Hwang S.J., Lee H.S., Kang T.W., Kim D.Y. (2006). Improved ferromagnetism of (Zn0.93 Mn0.07) O through rapid thermal annealing. Appl. Phys. Lett..

[18] Janisch R., Gopal P., Spaldin N.A. (2005). Transition metal-doped TiO_2_ and ZnO—Present status of the field. J. Phys. Condens. Matter.

[19] Gopal P., Spaldin N.A. (2006). Magnetic interactions in transition-metal-doped ZnO: An *ab initio* study. Phys. Rev. B.

[20] Zheng Y., Boulliard J.C., Demaille D., Bernard Y., Pétroff J.F. (2005). Study of ZnO crystals and Zn_1−x_M_x_O (M=Co, Mn) epilayers grown by pulsed laser deposition on ZnO(001) substrate. J. Cryst. Growth.

[21] Polyakov A.Y., Govorkov A.V., Smirnov N.B., Pashkova N.V., Pearton S.J., Ip K., Frazier R.M., Abernathy C.R., Norton D.P., Zavada J.M. (2004). Optical and magnetic properties of ZnO bulk crystals implanted with Cr and Fe. Mater. Sci. Semicond. Process..

[22] Dietl T., Ohno H., Matsukura F., Cibert J., Ferrand D. (2000). Zener model description of ferromagnetism in zinc-blende magnetic semiconductors. Science.

[23] Kim C.O., Kim S., Oh H.T., Choi S.-H., Shon Y., Lee S., Hwang H.N., Hwang C.-C. (2010). Effect of electrical conduction properties on magnetic behaviors of Cu-doped ZnO thin films. Phys. B Condens. Matter.

[24] Zheng J.H., Song J.L., Li X.J., Jiang Q., Lian J.S. (2011). Experimental and first-principle investigation of Cu-doped ZnO ferromagnetic powders. Cryst. Res. Technol..

[25] Shannon R.D. (1976). Revised effective ionic radii and systematic studies of interatomic distances in halides and chalcogenides. Acta Crystallogr. Sect. A.

[26] Keis K., Vayssieres L., Rensmo H., Lindquist S.-E., Hagfeldt A. (2001). Photoelectrochemical properties of Nano- to microstructured ZnO electrodes. J. Electrochem. Soc..

[27] Jeong T.S., Han M.S., Youn C.J., Park Y.S. (2004). Raman scattering and photoluminescence of As ion-implanted ZnO single crystal. J. Appl. Phys..

[28] Samanta K., Bhattacharya P., Katiyar R.S., Iwamoto W., Pagliuso P.G., Rettori C. (2006). Raman scattering studies in dilute magnetic semiconductor Zn_1−x_Co_x_O. Phys. Rev. B.

[29] Sharma P.K., Dutta R.K., Pandey A.C. (2009). Doping dependent room-temperature ferromagnetism and structural properties of dilute magnetic semiconductor ZnO:Cu^2+^ nanorods. J. Magn. Magn. Mater..

[30] Alim K.A., Fonoberov V.A., Balandin A.A. (2005). Origin of the optical phonon frequency shifts in ZnO quantum dots. Appl. Phys. Lett..

[31] Pradhan A.K., Zhang K., Loutts G.B., Roy U.N., Cui Y., Burger A. (2004). Structural and spectroscopic characteristics of ZnO and ZnO:Er^3+^ nanostructures. J. Phys. Condens. Matter.

[32] Cuscó R., Alarcón-Lladó E., Ibáñez J., Artús L., Jiménez J., Wang B., Callahan M.J. (2007). Temperature dependence of Raman scattering in ZnO. Phys. Rev. B.

[33] Wang H., Wang H.B., Yang F.J., Chen Y., Zhang C., Yang C.P., Li Q., Wong S.P. (2006). Structure and magnetic properties of Zn_1−x_Co_x_O single-crystalline nanorods synthesized by a wet chemical method. Nanotechnology.

[34] Xu C.X., Sun X.W., Zhang X.H., Ke L., Chua S.J. (2004). Photoluminescent properties of copper-doped zinc oxide nanowires. Nanotechnology.

[35] Fan H.J., Scholz R., Kolb F.M., Zacharias M., Gösele U., Heyroth F., Eisenschmidt C., Hempel T., Christen J. (2004). On the growth mechanism and optical properties of ZnO multi-layer nanosheets. Appl. Phys. A.

[36] Lupan O., Pauporté T., Viana B., Tiginyanu I.M., Ursaki V.V., Cortès R. (2010). Epitaxial electrodeposition of ZnO nanowire arrays on p-GaN for efficient UV-light-emitting diode fabrication. ACS Appl. Mater Interfaces.

[37] Wang X.B., Song C., Geng K.W., Zeng F., Pan F. (2007). Photoluminescence and Raman scattering of Cu-doped ZnO films prepared by magnetron sputtering. Appl. Surf. Sci..

